# Serum NGAL to Clinically Distinguish Cholangiocarcinoma from Benign Biliary Tract Diseases

**DOI:** 10.4061/2011/873548

**Published:** 2010-08-31

**Authors:** Kawin Leelawat, Siriluck Narong, Jerasak Wannaprasert, Surang Leelawat

**Affiliations:** ^1^Department of Surgery, Rajavithi Hospital, Bangkok 10400, Thailand; ^2^Faculty of Medicine, Rangsit University, Bangkok 10400, Thailand; ^3^Faculty of Pharmacy, Rangsit University, Pathumthani 12000, Thailand

## Abstract

*Aim*. To determine whether the serum level of NGAL can discriminate cholangiocarcinoma from benign biliary tract disease in patients. *Methods*. This study was performed according to a prospective-specimen-collection, retrospective-blinded-evaluation (PRoBE) design. A total of 50 cholangiocarcinoma and 50 benign biliary tract disease cases were randomly selected from a cohort of consecutive cases of biliary tract diseases. Their sera were measured for the levels of NGAL and the widely used serum cholangiocarcinoma marker, carbohydrate antigen 19-9 (CA19-9). *Results*. The serum CA19-9 and NGAL levels were significantly elevated in cholangiocarcinoma patients (CA19-9: *P* < .001, NGAL: *P* < .001). The area under the curve (AUC) of a receiver operating characteristic (ROC) curve analysis for the diagnosis of cholangiocarcinoma of CA19-9 and NGAL was 0.81 and 0.79, respectively. *Conclusion*. The diagnostic accuracy of serum NGAL and CA19-9 makes them good candidates for use as biomarkers to discriminate cholangiocarcinoma patients from benign biliary tract disease patients.

## 1. Introduction

Cholangiocarcinoma (CCA) is one of the most aggressive malignant tumors and is associated with local invasiveness and a high rate of metastasis [1, 2]. Most cholangiocarcinoma patients present with symptoms of biliary tract obstruction. However, many cases of benign biliary tract diseases also present with similar clinical symptoms [3]. In addition, this tumor typically grows along the bile duct without projecting outward from the bile ducts as a forming mass. Computed tomography (CT), ultrasound, and magnetic resonance imaging (MRI) are often inadequate to reveal this lesion [4]. Therefore, identification of tumor markers in the serum would be beneficial in the clinical management of this disease. 

Neutrophil gelatinase-associated lipocalin (NGAL) is a 25 kDa glycoprotein that belongs to the lipocalin superfamily and is characterized by its low molecular weight and its ability to bind to and transport a variety of hydrophobic ligands such as fatty acids, retinoids, and pheromones [5]. High NGAL expression has been reported in various cancers including colon cancer [6], breast cancer [7, 8], ovarian cancer [9], pancreatic cancer [10], and lung cancer [11]. Recently, we detected the expression of NGAL protein by immunohistochemistry in 24 paraffin-embedded cholangiocarcinoma specimens. We found that all cholangiocarcinoma specimens (24/24 cases) demonstrated the signaling for NGAL expression and intense expression of NGAL protein was noted in 75.0% (18/24 cases) of cholangiocarcinoma specimens [12]. In addition, we demonstrated that knockdown of NGAL gene expression by siRNA significantly suppressed *in vitro* invasiveness activity of cholangiocarcinoma cells [12]. 

Previous studies showed that the serum level of NGAL is significantly elevated in many types of cancers, including gastric cancer, nonsmall cell lung cancer, and hepatocellular carcinoma [13–15]. However, there are no studies on the serum levels of NGAL in cholangiocarcinoma patients. Therefore, the aim of the present study was to determine serum NGAL concentrations in these patients. In addition, the diagnostic sensitivity, specificity and area under the receiver operating characteristic (ROC) curves of serum NGAL for differentiating cholangiocarcinoma from benign biliary tract disease patients were defined and compared with the values of carbohydrate antigen 19-9 (CA19-9), which is the common marker for cholangiocarcinoma [16, 17]. To avoid selection bias, this study was designed as a nested case control study [18] that involved prospective collection of specimens before outcome ascertainment from a study cohort of patients with symptoms of biliary tract obstruction. The serum values of NGAL and CA19-9 were assayed in a blinded fashion in sera from randomly selected case patients (cholangiocarcinoma) and control subjects (benign biliary tract diseases) within the study cohort.

## 2. Materials and Methods

### 2.1. Study Design

This study was performed within the Department of Surgery, Rajavithi Hospital. The local ethics committee approved the study protocol. Sample size was determined on the basis of an expected area under the ROC curve of NGAL serum levels for the diagnosis of cholangiocarcinoma. There are no previous studies concerning the accuracy of serum NGAL in the diagnosis of cholangiocarcinoma. Therefore, we suggested that for the detection of serum NGAL to be clinically helpful, the area under the ROC curve (AUC) of serum NGAL should be higher than 0.70 in the diagnosis of cholangiocarcinoma. By using a significance level of 0.05 (two-sided) and a power of 0.95, we determined that a sample of 50 cholangiocarcinoma patients was required for the study [19]. This study was carried out according to a prospective-specimen-collection, retrospective-blinded-evaluation (PRoBE) design [18]. We prospectively included consecutive patients with symptoms of obstructive jaundice who had undergone ERCP, PTBD, or bile duct surgery during a period from June 2008 to March 2010. After the diagnosis of these patients was ascertained, we randomly selected 50 cases of cholangiocarcinoma and 50 cases of benign biliary tract diseases and assayed their values of serum NGAL. Because increased serum NGAL concentrations have also been found in renal insufficiency conditions [20], we therefore excluded patients with renal insufficiency.

### 2.2. Serum Collection and the Measurement of Serum Biochemistry

After receiving informed consent from the patients, 5 mL fasting peripheral venous blood was collected at the time before the procedures (ERCP, PTBD, or bile duct surgery). The serum was separated and stored at −78°C within 2 h. Assays for the serum levels of albumin, globulin, AST, ALT, total and direct bilirubin, alkaline phosphatase (ALP), and CA19-9 were conducted using routine automated methods in the Rajavithi Hospital Pathological Laboratory.

### 2.3. Measurement of Serum NGAL Levels

The serum levels of NGAL were measured using an enzyme-linked immunosorbent assay (ELISA) kit (R&D Systems, Minneapolis, MN). The diluted serum samples were added in duplicate to 96-well plates coated with NGAL antibody. After incubation at room temperature for two hours, the conjugated secondary antibody was added. The substrate solution was then added to the plates and incubated for one hour. Following termination of the reaction with the stop solution (1 M sulfuric acid), the optical density was measured at 450 nm using a spectrophotometric microplate reader. The concentration of NGAL in each sample was calculated from a standard curve. The scientist examining these serum specimens was blinded to the patient's diagnosis.

### 2.4. Statistical Analysis

Data are presented as the mean ± SD, unless otherwise stated. Comparisons between the quantitative variables were performed using the Mann-Whitney U or Student's *t*-test, as appropriate. Qualitative variables were reported as counts, and comparisons between independent groups were performed using Pearson Chi-squared tests. *P* values <.05 were considered significant. An ROC curve was generated by plotting the sensitivity against 1-specificity, and the area under the curve with 95% confidence intervals was calculated. The optimal cut-off points for NGAL were selected based on the ROC curve analysis. Sensitivity, specificity, positive predictive value, and negative predictive values were calculated using a 2 × 2 table of the collected data.

## 3. Results

### 3.1. Patient Characteristics

A total of 221 obstructive jaundice patients were enrolled. Then 50 cholangiocarcinoma and 50 benign biliary tract disease cases were randomly selected. The benign biliary tract diseases (control group) included intrahepatic duct stones (6 cases), common bile duct stones (42 cases), and benign bile duct strictures (2 cases). The cholangiocarcinoma cases included perihilar cholangiocarcinoma (36 cases), intrahepatic cholangiocarcinoma (9 cases), and middle and distal common bile duct cancer (5 cases). As shown in Table 1, no statistically significant differences in gender, age, serum BUN, creatinine, globulin, and ALT levels were identified among the data from the control patients when compared to the cholangiocarcinoma patients. However, the levels of serum bilirubin and ALP were significantly higher in cholangiocarcinoma patients than in the control patients.

### 3.2. Serum Levels of CA19-9 and NGAL in Cholangiocarcinoma and Benign Biliary Tract Diseases

**Figure 1:**
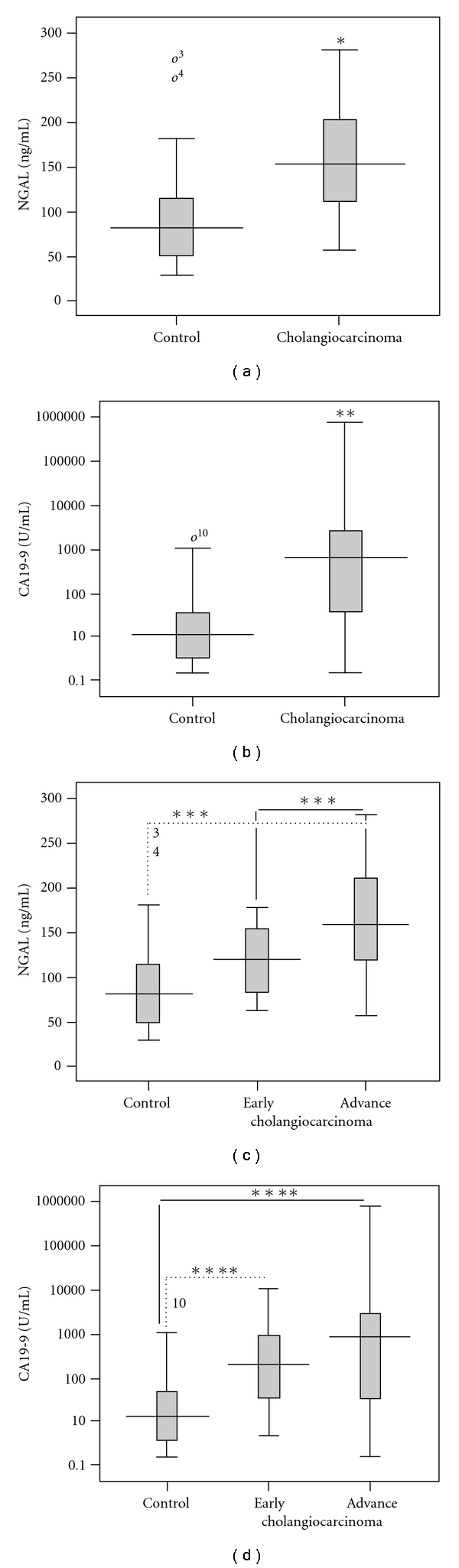
Serum levels of NGAL and CA19-9 in cholangiocarcinoma and control (benign biliary tract disease) patients. (a) Box plots comparing levels of NGAL and (b) CA19-9 between cholangiocarcinoma and control are illustrated. (c) Box plots comparing levels of NGAL and (d) CA19-9 between early and advanced stages of cholangiocarcinoma and control are illustrated. Levels of NGAL are presented as ng/mL, whereas CA19-9 is presented with the log data to accommodate the wide range. (*; Mann-Whitney U; *P* < .001 compared to control, **; Student's *t*-test; *P* < .001 compared to control, ***; Kruskal-Wallis test; *P* < .001 compared to control, ****; ANOVA; *P* < .001 compared to control).

The serum CA19-9 and NGAL levels were compared between the two disease groups. The median values of serum CA19-9 and NGAL levels in the control group were 10.6 U/mL (range: 0.60–3660.00 ng/mL) and 80.7 ng/mL (range: 29.71–261.07 ng/mL), respectively. The median values of serum CA19-9 and NGAL levels in the cholangiocarcinoma group were 636.7 U/mL (range: 0.6–71,000 U/mL) and 156.15 ng/mL (range: 56.49–280.77 ng/mL), respectively. As shown in Figures 1(a) and 1(b), serum CA19-9 and NGAL values were significantly higher in cholangiocarcinoma cases when compared to the control patients (CA19-9: *Mann-Whitney U test*; *P* < .001 and NGAL: *Mann-Whitney U test*; *P* < .001). 

Moreover, we also classified cholangiocarcinoma patients into two groups: early (TNM stage I and II; 8 patients) and advanced (TNM stage III and IV; 42 patients) stages. The data shown in Figure 1(c) demonstrate that the NGAL levels tended to increase according to the progression of cholangiocarcinoma. The serum NGAL values were significantly different between early and late stages of cholangiocarcinoma (ANOVA; *P* < .001). Although the serum CA19-9 values in the early and late stages of cholangiocarcinoma were significantly higher than in the controls (Kruskal-Wallis test; *P* < .001), the values were not significantly different between the early and late stages of cholangiocarcinoma (Figure 1(d)).

### 3.3. Correlation between NGAL, CA19-9, and Other Blood Chemistry

The correlation between the values of serum BUN, creatinine, albumin, AST, ALT, ALP, total bilirubin, CA19-9 and NGAL were investigated. As presented in Table 2, the level of serum NGAL was significantly correlated with serum CA19-9, albumin, and total bilirubin, although none of these parameters have a large Pearson's correlation coefficient (>0.7). We suggest that the significant correlation of these blood chemistries with serum NGAL is caused by the high number of samples used in this study.

### 3.4. Serum Levels of CA19-9 and NGAL for the Diagnosis of Cholangiocarcinoma

**Figure 2:**
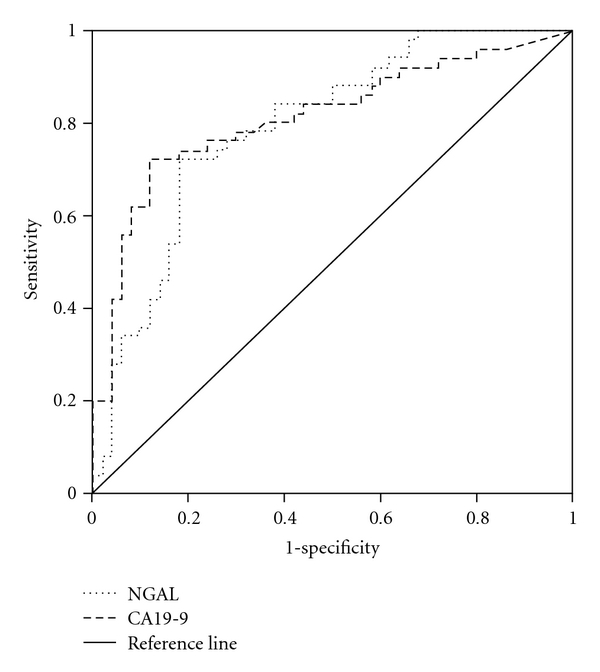
ROC curve analyses of NGAL and CA19-9 for the diagnosis of cholangiocarcinoma. The diagnostic accuracy of each biomarker, in terms of its sensitivity and specificity, is presented by receiver operating characteristic (ROC) curve analysis.

The diagnostic accuracy of serum CA19-9 and NGAL levels for differentiating cholangiocarcinoma from benign bile duct diseases was tested using an ROC curve analysis. The AUC of the ROC curve for serum CA19-9 and NGAL was 0.81 (95% CI 0.725–0.899) and 0.79 (95% CI 0.703–0.811), respectively (Figure 2). The sensitivity and specificity and positive and negative predictive values for selected cut-off points of CA19-9 and NGAL are presented in Table 3. The sensitivity of the combination of serum CA19-9 and NGAL in these patients is considerably better than either alone, with a combined sensitivity of 90%, and a combined specificity of 66% (slightly reduced).

## 4. Discussion

To our knowledge, this is the first study to assess serum NGAL and CA19-9 levels in patients with cholangiocarcinoma. Until now, there are two common tumor markers used for detecting cholangiocarcinoma, carcinoembryonic antigen (CEA), and CA19-9 [16]. CEA is nonspecific and can be elevated in the setting of other gastrointestinal or gynecologic malignancies or other bile duct pathologies, such as cholangitis and hepatolithiasis [21]. Our previous study demonstrated that the sensitivity and specificity of CEA as a marker for detecting cholangiocarcinoma are only 58.54% and 62.50%, respectively [17]. Therefore, we did not include the measurement of serum CEA in this study. Here, we demonstrated the statistically significant difference in the serum NGAL levels between cholangiocarcinoma patients and control patients (benign biliary tract diseases). When comparing the AUC of the ROC curve for CA19-9 and NGAL with a chance value equal to 0.5 (the worst value of AUC of ROC), both the AUC of the ROC for CA19-9 and NGAL were significantly higher than 0.5. This study demonstrated that the sensitivity of the combination of serum CA19-9 and NGAL for the detection of cholangiocarcinoma is promising (a combined sensitivity of 94%). This finding indicates that the serum values of CA19-9 and NGAL may be used as a biomarker to discriminate cholangiocarcinoma from benign biliary tract disease patients.

Although, in this study the significant differences between the cholangiocarcinoma and benign biliary tract diseases patients regarding to the values of serum AST, ALT, total bilirubin and albumin were identified. The Pearson's correlation coefficient between these serum biochemistries and serum NGAL was not high (<0.7). We suggest that the value of NGAL does not depend on these serum biochemistries. However, further studies, which classified the value of serum NGAL in the patients with different levels of these serum biochemistries should be performed.

The strength of the present study was in the implementation of PRoBE designs to avoid the problems of bias that may affect studies of the diagnostic test [18]. We collected serum from all obstructive jaundice patients before the diagnosis of cholangiocarcinoma or benign biliary tract diseases had been determined. This procedure ensured that biases related to differences in sample collection and handling would be avoided. Limitations of this design include the fact that the majority of the study participants were in advanced stages of cholangiocarcinoma. The number of patients with early-stage cholangiocarcinoma was small (*n* = 8). Further prospective multicentric studies, which should include a greater number of early-stage cholangiocarcinoma cases, need to be done to evaluate the roles of NGAL in early detection of cholangiocarcinoma, correlation with the grade of cholangiocarcinoma, and oncologic followup should be performed. In addition, this study was performed in the referral center, which has a high prevalence of cholangiocarcinoma cases. As a result, the findings may not be broadly applicable to other hospitals that typically have a lower volume of cholangiocarcinoma cases.

In conclusion, this study demonstrated that serum NGAL and CA19-9 levels are significantly elevated in cholangiocarcinoma patients. These markers have the potential to be used as tumor markers for the discrimination of cholangiocarcinoma patients from benign biliary tract disease patients.

## Figures and Tables

**Table 1:** Clinical characteristics of patients with benign biliary tract diseases (control) and cholangiocarcinoma.

	Control *N* = 50	Cholangiocarcinoma *N* = 50	*P value*
Age (yr)	61 ± 17	60 ± 9	.898
Sex (male : female)	15 : 35	19 : 31	.527
Albumin (mg/dL)	3.9 ± 0.73	3.0 ± 0.55	<.001
BUN (mg/dL)	14.7 ± 8.40	22.3 ± 28.74	0.107
Creatinine (mg/dL)	0.9 ± 0.75	1.1 ± 1.16	0.358
Globulin (mg/dL)	3.8 ± 0.69	4.1 ± 0.94	0.098
Total bilirubin (mg/dL)	2.7 ± 4.13	13.6 ± 11.56	<.001
AST (U/L)	63.4 ± 95.80	103.3 ± 77.77	0.001
ALT (U/L)	62.1 ± 80.13	56.8 ± 44.54	0.188
ALP (U/L)	253.5 ± 319.22	411.7 ± 312.60	<.001

**Table 2:** Pearson's correlation coefficients of NGAL, CA19-9, BUN, creatinine, albumin, total bilirubin, AST, ALT and ALP.

Pearson correlation	CA19-9	BUN	Creatinine	Albumin	Total bilirubin	AST	ALT	ALP
NGAL	0.26*	0.21	0.09	−0.61*	0.43*	0.22	−0.055	0.02
CA19-9	0.26*	0.09	0.08	−0.40*	0.40*	0.08	−0.04	0.17

*Statistically significant; *P* < .05.

**Table 3:** Performance of the biomarkers for the diagnosis of cholangiocarcinoma.

Tumor Markers (cut-off value)	Sensitivity (%) (95% CI)	Specificity (%) (95% CI)	LR+ (%) (95% CI)	LR- (%) (95% CI)
NGAL	100	26	1.35	—
(50 ng/mL)	(-)	(14–38)	(1.35–1.59)	(-)
NGAL	76	72	2.71	0.33
(110 ng/mL)	(64–88)	(60–84)	(1.69–4.35)	(0.20–0.56)
CA19-9	76	74	2.96	0.32
(35 U/mL)	(64–88)	(62–86)	(1.79–4.79)	(0.19–0.55)
CA19-9	72	86	5.14	0.33
(100 U/mL)	(60–84)	(76–96)	(2.53–10.44)	(0.21–0.51)
Combined markers*	90	66	2.65	0.15
	(82–98)	(53–79)	(1.78–3.94)	(0.06–0.36)

LR+; positive likelihood ratio, LR*‒*; negative likelihood ratio, CI; confidence interval. *; Combination of NGAL (cut-off value = 110 ng/mL) and CA19-9 (cut-off value = 100 U/mL).
